# *DPYD*, *TYMS* and *MTHFR* Genes Polymorphism Frequencies in a Series of Turkish Colorectal Cancer Patients

**DOI:** 10.3390/jpm8040045

**Published:** 2018-12-13

**Authors:** Arsalan Amirfallah, Gizem Calibasi Kocal, Olcun Umit Unal, Hulya Ellidokuz, Ilhan Oztop, Yasemin Basbinar

**Affiliations:** 1 Biomedical Center, Faculty of Medicine, University of Iceland, 101 Reykjavik, Iceland; arsalan.amirfallah@gmail.com; 2 Cell Biology Unit, Department of Pathology, Landspitali University Hospital, 101 Reykjavik, Iceland; 3 Department of Basic Oncology, Institute of Oncology, Dokuz Eylul University, 35350 Izmir, Turkey; gizem.calibasi@deu.edu.tr; 4 Personalized Medicine and Pharmacogenomics/Genomics Research Centre-BIFAGEM, 35350 Izmir, Turkey; 5 Bozyaka Education and Research Hospital, Division of Medical Oncology, Department of Internal Medicine, Health Science University, 35170 Izmir, Turkey; drolcun@hotmail.com; 6 Department of Preventive Oncology, Institute of Oncology, Dokuz Eylul University, 35350 Izmir, Turkey; hulya.ellidokuz@deu.edu.tr; 7 Department of Medical Informatics and Biostatistics, Faculty of Medicine, Dokuz Eylul University, 35350 Izmir, Turkey; 8 Department of Clinical Oncology, Faculty of Medicine, Dokuz Eylul University, 35350 Izmir, Turkey; ilhan.oztop@deu.edu.tr; 9 Department of Translational Oncology, Institute of Oncology, Dokuz Eylul University, 35350 Izmir, Turkey

**Keywords:** *DPYD*, *TYMS* and *MTHFR* genes, polymorphisms, pharmacogenetics, colorectal cancer

## Abstract

Fluoropyrimidine-based chemotherapy is extensively used for the treatment of solid cancers, including colorectal cancer. However, fluoropyrimidine-driven toxicities are a major problem in the management of the disease. The grade and type of the toxicities depend on demographic factors, but substantial inter-individual variation in fluoropyrimidine-related toxicity is partly explained by genetic factors. The aim of this study was to investigate the effect of *dihydropyrimidine dehydrogenase (DPYD), thymidylate synthase (TYMS), and methylenetetrahydrofolate reductase (MTHFR)* polymorphisms in colorectal cancer patients. Eighty-five patients who were administered fluoropyrimidine-based treatment were included in the study. The *DPYD*, *TYMS* and *MTHFR* polymorphisms were scanned by a next generation Sequenom MassARRAY. Fluoropyrimidine toxicities were observed in 92% of all patients. The following polymorphisms were detected: *DPYD* 85T>C (29.4% heterozygote mutants, 7.1% homozygote mutants), *DPYD* IVS 14+1G>A (1.2% heterozygote mutants), *TYMS* 1494del TTAAAG (38.4% heterozygote mutants, 24.7% homozygote mutants), *MTHFR* 677C>T (43.5% heterozygote mutants, 9.4% homozygote mutants) and *MTHFR* 1298A>C (8.2% heterozygote mutants, 2.4% homozygote mutants). A statistically significant association was demonstrated between *MTHFR* 677C>T and fluoropyrimidine-related toxicity (*p* value = 0.007). Furthermore, *MTHFR* 1298A>C was associated with hematopoietic toxicity (*p* value = 0.008). *MTHFR* polymorphisms may be considered as related factors of fluoropyrimidine toxicity and may be useful as predictive biomarkers for the determination of the colorectal cancer patients who can receive the greatest benefit from fluoropyrimidine-based treatments.

## 1. Introduction

Combination chemotherapy regimens, including fluoropyrimidine-based chemotherapy, mainly with 5-fluorouracil (5-FU), have been a standard treatment for colorectal cancer for many years [1]. Dihydropyrimidine dehydrogenase (DPD), thymidylate synthase (TS) and methylenetetrahydrofolate reductase (MTHFR) are important enzymes for 5-FU metabolism. Over 80% of 5-FU is metabolized by DPD in the liver. The conversion of 5-FU to the inactive dihydrofluorouracil (DHFU) through DPD is the rate-limiting step of the 5-FU catabolism [2,3]. TS catalyzes the conversion of deoxyuridine monophosphate (dUMP) to deoxythymidine monophosphate (dTMP), which is critical for DNA replication. The active metabolite of 5-FU forms a ternary complex with TS and 5,10-methylenetetrahydrofolate (5,10-MTHF), and leads to the lack of dTMP. The absence of dTMP leads to reduced DNA synthesis, dUMP misincorporation into DNA, and DNA damage (double and single-strand breaks), followed by cell apoptosis [4,5]. MTHFR plays a role in the metabolism of folate. The substrate for MTHFR—5,10-MTHF—is used for the conversion of dUMP to dTMP by TS, whereas the product of 5-methyltetrahydrofolate is the methyl donor for the synthesis of methionine and *S*-adenosylmethionine in methylation reactions [6]. Studies have showed that 5-FU-based therapies prolong survival and reduce the risk of relapse. Clinically, colorectal cancer patients treated with combination regimes, including 5-FU, experience unavoidable treatment-related toxicities such as diarrhea, stomatitis, nausea, mucositis, myelosuppression, and hand-foot syndrome [1]. The grade and type of the toxicities depend on demographic factors such as sex, age, fluoropyrimidine application dose and mode of administration [7,8]. Substantial inter-individual variation in fluoropyrimidine-related toxicity is partly explained by genetic factors. The polymorphisms in the *dihydropyrimidine dehydrogenase (DPYD)*, *thymidylate synthase (TYMS)*, and *methylenetetrahydrofolate reductase (MTHFR)* genes have been reported as the reason for severe adverse reactions [9]. The aim of this study was to investigate the effect of *DPYD*, *TYMS* and *MTHFR* polymorphisms on the observed toxicities associated with fluoropyrimidine-based treatment in colorectal patients.

## 2. Materials and Methods

### 2.1. Study Population and Setting

Eighty-five colorectal cancer patients who were treated with fluoropyrimidine-based chemotherapy regimens between 2011 and 2013 at the Dokuz Eylul University Hospital were enrolled in this retrospective study. Informed consent was obtained from all patients. The study was based on routine blood material approved by the Non-Invasive Research Clinical Research Ethics Committee of Dokuz Eylul University School of Medicine (No: 2012/05-08). All patients had to fulfill the following inclusion criteria for the study: (i) diagnosed with a colorectal tumor and had fluoropyrimidine-based chemotherapy in Dokuz Eylul University Hospital, (ii) adequate hematological and cardiac status, (iii) sufficient biological material for genotyping analysis. Clinical data were obtained from standardized patient records. 

### 2.2. Demographic Data

The characteristics of the 85 colorectal cancer patients treated with fluoropyrimidine-based agents are shown in Table 1. The mean age was 58.88 (range 20–81). The majority of the patients were men (67.1%). The tumor location was predominantly the colon (67.1%) and rectum (32.9%). The histopathological type and the stage of the tumors were mostly observed as adenocarcinoma (91%) and stage IV (60%), respectively. The staging of the tumor was conducted when a patient was first diagnosed, before any treatment was given, according to the American Joint Committee on Cancer’s (AJCC) Cancer Staging 6th edition 2002 TNM grading system [10].

### 2.3. Toxicity Evaluation

The toxicities associated with fluoropyrimidine-based treatment (such as gastrointestinal, hematopoietic, hair-skin toxicities and hand-foot syndrome) during the first two cycles of chemotherapy were evaluated at each clinic visit by anamnesis, physical examination, and the results of hematological, biochemical and urine tests. All adverse drug reactions and toxicities in the study group were recorded and graded according to the Common Terminology Criteria for Adverse Events v4.0 (CTCAE). Mucositis, ascites, colitis, diarrhea, dry mouth, gastritis, nausea and vomiting were grouped as gastrointestinal toxicity; anemia, febrile neutropenia and leukocytosis were grouped as hematopoietic toxicity; alopecia, dry skin, nail discoloration, nail loss, nail ridging, purpura, skin hyperpigmentation, skin hypopigmentation and skin ulceration were grouped as hair and skin toxicity; and hand-foot syndrome was placed in a discrete group. Grade 1 reflected a mild reaction, grade 2 a moderate reaction, grade 3 a reaction that was severe or medically significant but not immediately life-threatening, and grade 4 indicated a life-threatening adverse drug reaction. 

### 2.4. Genotyping

Patients’ peripheral blood samples were collected in EDTA-supplemented tubes. Samples were stored at −80 °C until testing. Genomic DNA was extracted using the Invisorb Spin Blood Mini Kit (Invitek, Berlin, Germany). Single nucleotide polymorphism (SNP) genotyping of the *DPYD*, *TYMS* and *MTHFR* genes was performed with a MassARRAY platform (Sequenom Inc., San Diego, CA, USA), which relies on the natural molecular weight differences of DNA bases. This high-throughput platform combines the sensitivity of the polymerase chain reaction (PCR) and the accuracy of matrix-assisted laser desorption-ionization time-of-flight mass spectrometry (MALDI-TOF MS) [11]. PCR and iPLEX (Sequenom Inc., San Diego, CA, USA )single base extension primers for the multiplexed assays were designed with the MassARRAY^®^ Designer software (Sequenom Inc.). Initial-locus-specific PCR reactions and locus-specific primer extension reactions (iPLEX assay) with oligonucleotide primers were performed to amplify specific polymorphic sites. MALDI-TOF mass spectrometry was used to distinguish the differences in the masses of allele-specific primer extension products for *DPYD* IVS 14+1G>A (DPYD*2A allele), *DPYD* 85T>C (DPYD*9A allele), *DPYD* 1679T>G/T (DPYD*13A allele), *DPYD* 2846A>T, *TYMS* 1494 ins/del, *MTHFR* 677C>T and *MTHFR* 1298A>C polymorphisms. The obtained data were analyzed using the MassARRAY Typer 4.0 Analyzer software (Sequenom Inc.).

### 2.5. Statistical Analysis

The Statistical Package for the Social Sciences (SPSS) (Version 21.0; SPSS, Inc., Chicago, IL, USA) program was used for statistical analysis. Gender, tumor location, histologic type of tumor, stage of tumor, observed toxicities, and frequency of polymorphisms defined as percentage of patients were analyzed. Toxicities were grouped by four classes, including gastrointestinal toxicity (diarrhea, mucositis, nausea), hematopoietic toxicity (myelosuppression), skin-hair toxicity (dry, cracking, peeling skin, hyperpigmentation, alopecia, hair thinning) and hand-foot syndrome. The Hardy-Weinberg equilibrium (HWE) of genotypes was analyzed by chi square test. The genotypes were discussed in relation to the previously published frequencies and Database of Single Nucleotide Polymorphisms (dbSNP) [12]. The chi- square test was used to determine the association of *DPYD*, *TYMS* and *MTHFR* polymorphisms with the existence of toxicity. A *p* value < 0.05 was considered as significant.

## 3. Results

### 3.1. Patients’ Toxicity Types and Grades

Based on the Common Terminology Criteria for Adverse Events v4.0 (CTCAE), 91.8% (*n* = 78) of the patients suffered from toxicities (Grade 1: 32.9%, Grade 2: 22.4%, Grade 3: 29.4, Grade 4: 7.1%). According to the toxicity classification, hematopoietic toxicity (54.1%) (Grade 1: 16.5%, Grade 2: 9.4%, Grade 3: 21.2%, Grade 4: 7.1%), hand-foot syndrome (21.2%) (Grade 1: 9.4%, Grade 2: 7.1%, Grade 3: 4.7%), gastrointestinal (12.9%) (Grade 1: 5.9%, Grade 2: 5.9%, Grade 3: 1.2%) and skin-hair toxicities (3.5%) (Grade 1: 1.2%, Grade 3: 2.4%) were observed (Table 2).

### 3.2. Patient Genotypes

The polymorphism distribution is presented in Table 3. *DPYD* 85T>C (mutant allele frequency 0.22; polymorphism frequencies; 29.4% heterozygote mutants, 7.1% homozygote mutants), *DPYD* IVS 14+1G>A (mutant allele frequency 0.01; polymorphism frequencies; 1.2% heterozygote mutants, 0% homozygote mutants), *TYMS* 1494 del TTAAAG (mutant allele frequency 0.44; polymorphism frequencies; 38.4% heterozygote mutants, 24.7% homozygote mutants), *MTHFR* 677C>T (mutant allele frequency 0.31; polymorphism frequencies; 43.5% heterozygote mutants, 9.4% homozygote mutants) and *MTHFR* 1298A>C (mutant allele frequency 0.09; polymorphism frequencies; 8.2% heterozygote mutants, 2.4% homozygote mutants). Determined SNPs were in Hardy-Weinberg Equilibrium (*p* values were 0.955, 0.208, 0.049, 0.895 and 0.001 for *DPYD* IVS14+1G>A, *DPYD* 85T>C, *TYMS* 1494 del TTAAAG, *MTHFR* 677C>T and *MTHFR* 1298A>C respectively). The *DPYD* 1679T>G/T and *DPYD* 2846A>T polymorphisms were not detected in our group.

### 3.3. Impact of Gene Polymorphisms on Toxicity

At least one of four toxicity groups (gastrointestinal, hematopoietic, skin-hair toxicity and hand-foot syndrome) was observed in all patients with the *MTHFR* 677C>T polymorphism (heterozygote/homozygote) (*p* value, 0.007) (Table 4). However, there was no association between *MTHFR* 677C>T polymorphism (heterozygote/homozygote) and a specific toxicity group. The existence of the *MTHFR* 1298A>C polymorphism (heterozygote/homozygote) was associated with hematopoietic toxicity (*p* value, 0.008) (Table 5). Statistically significant associations could not be demonstrated between *DPYD*, *TYMS* polymorphisms and fluoropyrimidine-driven toxicities.

## 4. Discussion

The most widely used chemotherapy drugs in the treatment of colorectal cancer patients are 5-FU and capecitabine (pro-drug of 5-FU). Thymidylate synthase (TS), dihydropyrimidine dehydrogenase (DPD) and methylenetetrahydrofolate reductase (MTHFR) enzymes play important roles in the metabolic pathway of this chemotherapeutic [13,14,15]. In this study, common polymorphisms in the *DPYD*, *TYMS* and *MTHFR* genes that encode the DPD, TS and MTHFR enzymes, were investigated to understand their effects on fluoropyrimidine-related toxicity in Turkish colorectal cancer patients. Genomic DNA from eighty-five colorectal patients was analyzed for *DPYD* IVS14+1G>A (DPYD*2A allele), *DPYD* 85T>C (DPYD*9A allele), *DPYD* 1679T>G/T (DPYD*13 allele), *DPYD* 2846A>T, *TYMS* 1494 del TTAAAG, *MTHFR* 677C>T and *MTHFR* 1298A>C polymorphisms using a MassARRAY platform.

In the study group, *DPYD* IVS 14+1G>A (DPYD*2A allele), *DPYD* 85T>C (DPYD*9A allele), *TYMS* 1494 del TTAAAG, *MTHFR* 677C>T and *MTHFR* 1298A>C polymorphisms were detected; although *DPYD* 1679T>G/T (DPYD*13A allele) and *DPYD* 2846A>T polymorphisms were not. The distribution of polymorphisms and allelic frequencies are presented in Table 3.

**Figure 1:**
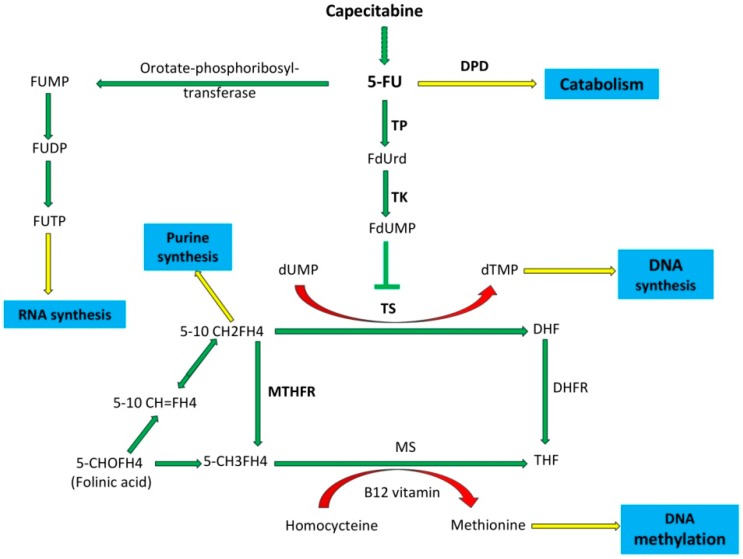
Metabolism pathway of capecitabine and 5-fluorouracil (5-FU). Abbreviations: 5-fluoro uracil (5-FU), Thymidine phosphorylase (TP), 5-fluorodeoxyuridine (FdUrd), thymidine kinase (TK), 5-10 methylene-tetrahydrofolate (5-10 CH2FH4), dihydrofolate (DHF), 5-methyltetrahydrofolate (5-CH3FH4), methionine synthase (MS), tetrahydrofolate (THF), dihydrofolate reductase (DHFR), 5-formyltetrahydrofolate (folinic-acid) (5-CHOFH4), and 5-10 methenyltetrahydrofolate (5-10 CH=FH4).

The DPD enzyme functions as a rate-limiting enzyme in the catabolism of 5-FU. The DPD enzyme catalyzes the conversion of 5-FU into 5-fluoro-5, 6-dihydrouracil (5-FUH2), 5-FUH2, is then converted to fluoro-beta-ureidopropionate (FUPA) and subsequently to fluoro-beta-alanine (FBAL) by dihydropyrimidinease (DPYS) and beta-ureidopropionase (UPB1) enzymes, respectively (Figure 1). Single nucleotide polymorphisms in the *DPYD* gene are responsible for low levels of DPD enzyme. This insufficient production of DPD results in excess drug accumulation and toxicity due to the inefficient catabolism of the drug. Different *DPYD* polymorphisms and polymorphism-driven functional defects in this enzyme could lead to abnormal 5-FU metabolism and a wide variety of clinical manifestations. A number of studies have reported that patients with a *DPYD* mutated allele suffered from severe toxicity after fluoropyrimidine-based treatments. *DPYD* 85T>C polymorphism caused the formation of a DPYD*9A allele with the conversion of Cys29Arg. In our study, the genotype frequency of the *DPYD* 85T>C polymorphism was 29.4% as a heterozygote mutation and 7.1% as a homozygote mutation. Although some groups have reported that the *DPYD* 85T>C polymorphism is predictive of fluoropyrimidine-related diarrhea, hand-foot syndrome and possibly ocular toxicity, no association was found between the *DPYD* 85T>C polymorphism and toxicity in our group [16,17].

The *DPYD* IVS14+1G>A splice-site transition results in a 165-bp deletion (corresponding to exon 14) in the *DPYD* mRNA and forms the DPYD*2A allele. The catalytic activity of the new truncated protein product is extremely low. The polymorphism analysis of the frequency showed that the IVS14+1G>A polymorphism was the most common among the cancer patients suffering from severe 5-FU-associated toxicity. Several reports have been published regarding the IVS14+1G>A splice-site transition frequency in subjects of different ethnic backgrounds and appears most commonly in Caucasian people. However, in our group, this polymorphism was the second most common, with 1.2%, among the *DPYD* polymorphisms [13,15].

*DPYD* 1679T>G/T and *DPYD* 2846A>T have consistently been associated with fluoropyrimidine toxicity. *DPYD* 1679T>G substitution results in the DPYD*13 allele due to the the change in I560S, and *DPYD* 2846A>T causes D949V to change. Both of them have been reported to be associated with low enzyme activity but have been observed as rare polymorphisms. In this study, the *DPYD* 1679T>G/T and *DPYD* 2846A>T polymorphisms were not detected [18,19].

In this report, the distributions and allele frequencies of the various *DPYD* polymorphisms (IVS14+1G>A, 85T>C, 1679T>G/T, 2846A>T) were found to be similar to previously published reports. Our results were similar to Caucasian/European-based studies on *DPYD* 85T>C and *DPYD* IVS14+1G>A polymorphisms. However, for *DPYD* 1679T>G/T and *DPYD* 2846A>T polymorphisms, our results were compatible with Asian-based studies. The allelic frequencies of all *DPYD* polymorphisms in this study were similar to global minor allelic frequency (MAF) data from dbSNP [15]. In contrast to the similarity of the genetic distributions, a statistically significant association could not be demonstrated between *DPYD* polymorphisms and fluoropyrimidine toxicities. Ethnic variations can change the clinical presentation of the disease and the pharmacokinetic/dynamic processes of the drugs. There are no adequately published data from a large Turkish population that investigate this in this respect. For this reason, large population-based studies are urgently needed to successfully manage the disease. Various enzymes (e.g., cytidine deaminase or CDA) and different polymorphisms also have roles in 5-FU metabolism [20]. These different polymorphisms should also be checked for their association with toxicity.

The primary mechanism of action of 5-FU is TS inhibition with fluorodeoxyuridine monophosphate (FdUMP), an active metabolite of 5-FU. FdUMP, TS and 5-10-methylenetrahydrofolate (MTHF) form an inactive triple complex (Supplementary Figure S1). This inhibition leads to the accumulation of deoxy-uridine-monophospate (dUMP) and the depletion of deoxy-thymidine-monophosphate (dTMP), which lead to the inhibition of DNA synthesis. The 5-FU can also inhibit RNA synthesis in a pathway that involves 5-fluorouridine monophosphate (FUMP) and subsequent conversion to 5-fluorouridine triphosphate (FUTP) via 5-fluorouridine diphosphate (FUDP). The optimal formation of FdUMP/TS/MTHF inactive triple complex requires a high MTHF level regulated by the methylenetetrahydrofolate reductase enzyme (MTHFR) on the catabolism process of 5-FU. Therefore, both TS and MTHFR activity are thought to be the determining factor for the 5-FU clinical response [4].

The presence of *TYMS* 1494 del TTAAAG at the 3′-UTR of gene (an insertion/deletion of a 6-bp sequence) is known to influence TS expression by leading to the formation of a less stable mRNA that will be more susceptible to degradation and have a decreased TS expression. *TYMS* 1494 del TTAAAG has been associated with both fluoropyrimidine-related toxicity and increased clinical responses [20]. Moreover, in most of the studies that were conducted with Caucasian (German, French, and Italian) or Asian (Chinese, Japanese) populations (52–60%), no significant association was found between the *TYMS* 1494 del TTAAAG polymorphism and fluoropyrimidine toxicity [21]. Although the *TYMS* 1494 del TTAAAG polymorphism was detected in 63.1% of all patients (38.4% heterozygote mutants, 24.7% homozygote mutants), a statistically significant association could not be demonstrated between the *TYMS* 1494 del TTAAAG polymorphism and the 5-FU toxicities. The potential importance of *TYMS* polymorphisms on fluoropyrimidine toxicity is still controversial. For accurate and decisive clinical results regarding *TYMS*, further systematic studies with large patient populations are required.

MTHFR is another key enzyme for 5-FU metabolism. It catalyzes the conversion of 5,10 methylenetetrahydrofolate (5,10-MTHF) to 5-methylenetetrahydrofolate (5-MTHF) [22]. Despite finding more than 60 germline polymorphisms in *MTHFR* [23], only *MTHFR* 677C>T, *MTHFR* 1298A>C, which are nonsynonymous SNPs, reduce *MTHFR* enzyme activity [24].

In our group, the *MTHFR* 677C>T frequency (43.5% heterozygote mutants, 9.4% homozygote mutants) was similar to the Caucasian-based studies, and it is evident that this polymorphism is not common in Middle Eastern or African populations (Supplementary Table S1) [25]. The allelic frequency of *MTHFR* 677C>T was determined to be 0.31 and was close to the global minor allelic frequency data from dbSNP [12]. 

The *MTHFR* 1298A>C polymorphism was quite rare in our group (8.2% heterozygote mutants, 2.4% homozygote mutants). The frequency of this polymorphism is higher in Caucasian, Asian and African-based populations than in our study (Supplementary Table S1). The frequency of both polymorphisms varies significantly between regions and ethnic groups [22,25]. The allelic frequency of the *MTHFR* 1298A>C polymorphism was determined to be 0.09, which is not even close to the global minor allelic frequency data (0.25) for dbSNP [12].

Although the *MTHFR* enzyme, which was involved in the 5-FU metabolic pathway, has been studied by several groups, the presented results have been controversial regarding the role of the *MTHFR* 677C>T and *MTHFR* 1298A>C polymorphisms on fluoropyrimidine toxicity in colorectal cancer patients [7]. 

In our study, a statistically significant association was demonstrated between the *MTHFR* 677C>T polymorphism (heterozygous/homozygous) and fluoropyrimidine toxicity (*p* value 0.009) (Table 3). Our results were compatible with the literature. Afzal et al. and Toffili et al. reported that the *MTHFR* 677C>T polymorphism was associated with fluoropyrimidine-related toxicity in colorectal and breast cancer patients, respectively, without claiming a specific toxicity type [5,26]. Lu et al. demonstrated an association between the *MTHFR* 677C>T polymorphism and a higher frequency of nausea and vomiting in gastric cancer patients [27]. Noor et al. found a significant correlation of the *MTHFR* C677T polymorphism with increased tumor response to 5-FU and developing grade 3 or 4 neutropenia, diarrhea, and mucositis in colorectal cancer patients [28]. In contrast to these reports and our results, Capitain et al. found no significant association in *MTHFR* 677C>T mutant patients in terms of 5-FU toxicity in patients with advanced colorectal cancer [29].

We found an association between the *MTHFR* 1298A>C polymorphism and hematopoietic toxicity. In the literature, the same contradicting results have been shown for the *MTHFR* 1298A>C polymorphism. Some researchers, such as Afzal et al., have reported no association between the *MTHFR* 1298A>C polymorphism and toxicity [5]. However, several researchers have reported that the *MTHFR* 1298A>C polymorphism had importance for the prediction of toxicity or the type of toxicity. Thomas et al. showed that *MTHFR* 1298A>C polymorphism is predictive of diarrhea or mucositis upon fluoropyrimidine administration as a single agent in rectal cancer patients [4]. Loganayagam et al. reported that both the *MTHFR* 677C>T and *MTHFR* 1298A>C polymorphisms were associated with hand–foot syndrome in gastrointestinal and colorectal patients [20]. Diarrhea, mucositis and hand–foot syndrome were also observed as fluoropyrimidine-related toxicities in the presence of the *MTHFR* 1298A>C polymorphism. However, there have been no reports of hematopoietic toxicity in the presence of the *MTHFR* 1298A>C polymorphism in colorectal patients [5]. In our study, the patient group who used capecitabine was small. Therefore, we could not carry out further statistical analysis in regard to capecitabine. The same analysis should be made with a larger population of patients using capecitabine.

The *MTHFR* 677C>T polymorphism shows a wide regional and ethnic diversity, with a genotype frequency from 12.6% to 44.5% for heterozygote mutations and from 0% to 13.2% for homozygote mutations. The frequencies of the homozygote (TT) or heterozygote (CT) genotypes among Caucasian/European populations are higher than in Asian, Middle Eastern and African populations. In this Turkish population-based study, the frequency results are close to those for Caucasians/Europeans. On the other hand, the *MTHFR* 1298A>C polymorphism does not present as much population diversity. Its frequency is more uniform within the studied groups. Table 4 shows the frequencies of polymorphisms with the heterozygous and homozygous genotypes, according to different ethnic populations.

Although 5-FU metabolism-related polymorphisms have been studied by several groups, the presented results have been controversial regarding the effects of the polymorphisms on fluoropyrimidine toxicity in colorectal cancer patients. 

The study groups generally included mixed ethnic populations, and ethnic variation affected the frequency and type of genetic mutations. Pharmacogenetic studies require a certain level of homogeneity in the selection of patients and therapies.

The weakness of this study was the limited number of patients. The association of the *MTHFR* polymorphisms used in this study with fluoropyrimidine-based chemotherapy-related toxicities in colorectal cancer patients needs to be confirmed in larger prospective study populations with a uniform chemotherapy regimen.

## 5. Conclusions

Fluoropyrimidine-based chemotherapeutic agents, 5-FU or capecitabine, have remained the mainstay of chemotherapeutic regimens for colorectal and gastrointestinal cancers in both metastatic and adjuvant settings. Therefore, it is important to identify decisive parameters that may help in the prognostic or predictive assessment of disease and drug toxicity. A drug response is a complex trait that involves many proteins. From a biological point of view, it is expected that different metabolic routes compete and that the effect of a polymorphism on a drug metabolism pathway can be altered by other polymorphisms [7]. Investigation into the role of polymorphisms influencing the fluoropyrimidine metabolism appears to be a promising area for further research. 

Although our study population was small, according to our results, MTHFR *677C>T* can be used for the prediction of fluoropyrimidine-based chemotherapy toxicities and *MTHFR 1298A>C* polymorphisms for the prediction of hematopoietic toxicities in colorectal cancer patients.

All of our findings and the supporting or opposing reports from various groups need to be validated in larger population-based cohorts, and the results should be compared with the toxicities of both single agent-based or combination-based fluoropyrimidine therapies.

## Figures and Tables

**Table 1:** Patient characteristics of the study cohort.

Demographic Details		Patients %
Gender *n* (%)	Male	67.1
	Female	32.9
Age	Range	20–81
	Mean	58.88
Primary Tumor Site	Colon	67.1
	Rectum	32.9
Histopathological Type	Adenocarcinoma	91
	Other	9
Stage of Tumor	I	0
	II	9.4
	III	30.6
	IV	60

**Table 2:** Major types of toxicity in patients receiving 5-fluorouracile-based chemotherapy in the first four cycles of treatment.

Toxicity Types	Grade of Toxicities, %			
	Grade 1	Grade 2	Grade 3	Grade 4
Gastrointestinal toxicity	5.9	5.9	1.2	-
Hematopoietic toxicity	16.5	9.4	21.2	7.1
Hair and skin toxicity	1.2	-	2.4	-
Hand-foot Syndrome	9.4	7.1	4.7	-

**Table 3:** Genotyped polymorphisms and allelic frequencies of detected polymorphisms among all patients.

Polymorphism	SNP no	Amino Acid Change	Genotype Frequency %			Allelic Frequency	
			wt/wt ^1^	wt/mut ^2^	mut/mut	wt	mut
DPYD*2A (IVS14+1G>A)	rs3918290	Exon skipping- Exon 14	GG (98.8)	AG (1.2)	AA (0)	0.99	0.01
DPYD*9A 85T>C	rs1801265	Cys29Arg- Exon 2	TT (63.5)	TC (29.4)	CC (7.1)	0.78	0.22
DPYD*13 1679T>G/T	rs55886062	I560S- Exon 13	AA (100)	AC (0)	CC (0)	1	0
*DPYD* 2846A>T	rs67376798	D949V- Exon 22	AA (100)	AT (0)	TT (0)	1	0
*TYMS* 1494 del TTAAAG	rs34489327	6 bp deletion-3′-UTR region	(-;-) (36.5)	(-;TTAAAG) (38.8)	(TTAAAG;TTAAAG) (24.7)	0.56	0.44
*MTHFR* 677C>T	rs1801133	A222V- Exon 4	CC (47.1)	CT (43.5)	TT (9.4)	0.69	0.31
*MTHFR* 1298A>C	rs1801131	E429A- Exon 7	AA (89.4)	AC (8.2)	CC (2.4)	0.91	0.09

^1^ wt: wild type allele; ^2^ mut: mutant allele.

**Table 4:** Correlation of *MTHFR* 677C>T polymorphism with the presence of fluoropyrimidine-driven toxicities.

*MTHFR* 677C>T	Toxicity (*n*)		Risk Ratio		95% CI		*p* Value	
	+	−						
Het (CT) + Hom (TT)	45 ^1^	0	1.18		(1.03–1.34)		0.007	
Wild Type (CC)	34	6						

^1^ Heterozygous (*n* = 37) + Homozygous mutant (*n* = 8). CI: confidence interval.

**Table 5:** Correlation of *MTHFR* 1298A>C polymorphism with presence of fluoropyrimidine-driven hematopoietic toxicity.

*MTHFR* 1298A>C	Hematopoietic Toxicity (*n*)		Risk Ratio		95% CI		*p* Value	
	+	−						
Het (AC) + Hom (CC)	9 ^1^	0	1.86		(1.50–2.32)		0.008	
Wild Type (AA)	37	32						

^1^ heterozygous (*n* = 2) + Homozygous mutant (*n* = 7).

## Supplementary Materials

The following are available online at http://www.mdpi.com/2075-4426/8/4/45/s1, Figure S1: Catabolism of capecitabine and 5-fluorouracil (5-FU), Table S1: Genotype frequencies of *MTHFR* 677C >T and 1298A>C polymorphisms in various ethnic groups including our study.
