# Supplementary material for: DPYD, TYMS and MTHFR Genes Polymorphism Frequencies in a Series of Turkish Colorectal Cancer Patients
Source: J Pers Med. 2018 Dec 13;8(4):45. doi: 10.3390/jpm8040045 (PMC6313617; doi:10.3390/jpm8040045)
Supplement: Supplementary file 1 [file jpm-08-00045-s001.pdf]

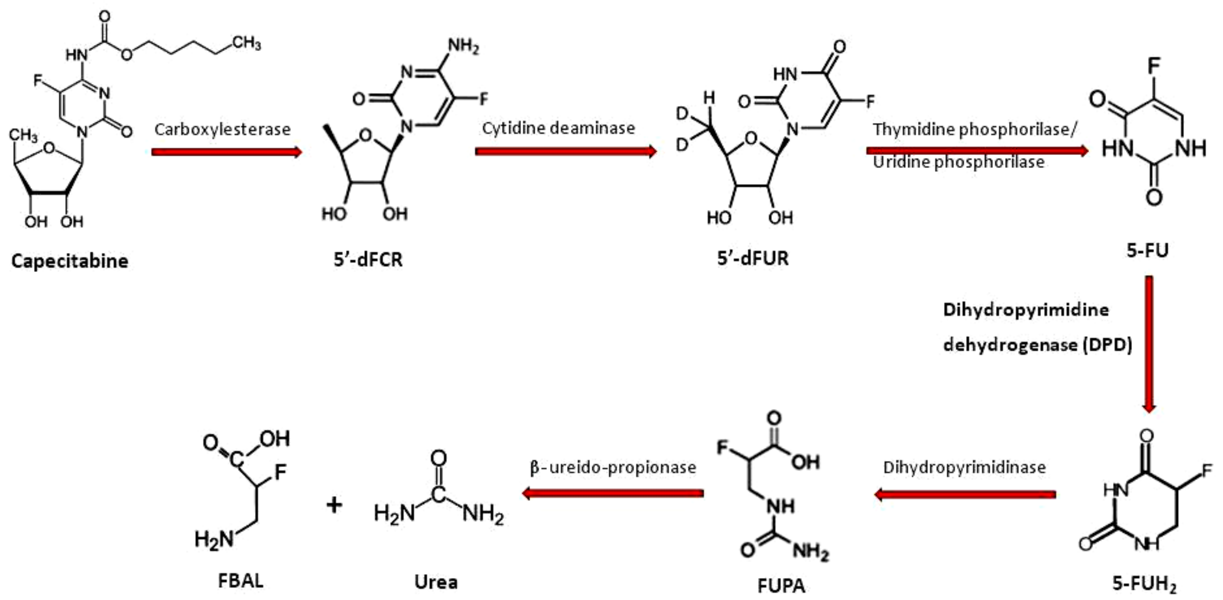

Supplementary Figure S1. Catabolism of capecitabine and 5-fluorouracil (5-FU)

5′-dFCR, 5′-deoxy-5-fluorocytidine; 5′-dFUR, 5′-deoxy-5-fluorouridine; 5-FU, 5-fluoro uracil; 5-FUH2, 5-fluorodihydrouracil; FUP, fluoro-β-ureidopropionate; FBAL, fluoro-β-alanine.

Supplementary Table S1. Genotype frequencies of MTHFR 677C >T and 1298A>C polymorphisms in various ethnic groups including our study.

	MTHFR 677C >T, Genotype frequency %		
	**wt/wt** [1]	**wt/mut** [2]	**mut/mut**
This study/ Turkish	**47.1**	**43.5**	**9.4**
Caucasian/European	42.3	44.5	13.2
Asian	68.2	28.3	3.5
Middle Eastern	72.8	24.7	2.5
African	87.4	12.6	0

	MTHFR 1298A>C, Genotype frequency %		
	**wt/wt**	**wt/mut**	**mut/mut**
This study/ Turkish	**89.4**	**8.2**	**2.4**
Caucasian/European	44.1	42.5	10.7
Asian	67.2	31.4	1.4
Middle Eastern	46.8	43.1	10.2
African	61.4	34.2	4.4
